# Supplementary material for: Precise transcriptional control of cellular quiescence by BRAVO/WOX5 complex in Arabidopsis roots
Source: Mol Syst Biol. 2021 Jun 16;17(6):e9864. doi: 10.15252/msb.20209864 (PMC8207686; doi:10.15252/msb.20209864)
Supplement: Supplementary file 2 — Appendix [file MSB-17-e9864-s001.pdf]

APPENDIX

TABLE OF CONTENTS

Alleviation model 1

Activation model 3

Complex formation model 4

Stationary solutions 6

Supplementary Figures and Tables 11

Alleviation model

For the WT genotype, the alleviation model reads (see Material and Methods):

$$\frac{dB}{dt} = P_B(B,W) - d_B B$$

$$P_B(B,W) = \alpha \frac{1+\varepsilon_B (K_B B)^2}{1+(K_B B)^2} \frac{1+\varepsilon_W (K_W W)^2}{1+(K_W W)^2}$$

$$\frac{dW}{dt} = P_W(B,W) - d_W W$$

$$P_W(B,W) = \gamma \frac{W_0^2}{W_0^2 + W^2 \left(\frac{B_0^2}{B^2+B_0^2} B_1 + 1 \right)^2}$$

By redefining time as $\tau = t d_B$ defining the new nondimensinal variables $b = K_B B, w = K_W W$ and parameters $\tilde{\alpha} = \alpha K_B / d_B$, $\tilde{\gamma} = \gamma K_W / d_W$, $b_0 = B_0 K_B$, $w_0 = W_0 K_W$, we can write a nondimensional version of the model as:

$$\frac{db}{d\tau} = \tilde{\alpha} \frac{1+\varepsilon_B b^2}{1+b} \frac{1+\varepsilon_W w^2}{1+w^2} - b$$

$$\frac{dw}{d\tau} = \frac{d_W}{d_B}\left(\tilde{\gamma}\frac{w_0^2}{w_0^2 + w^2\left(\frac{b_0^2}{b^2 + b_0^2}B_1 + 1\right)^2} - w\right)$$

With this nondimensional model we computed the distribution of promoter fold-changes when several parameters are allowed to change all at once.

For clarity, here we show how to compute the mutants with the full, dimensional model.

For the *wox5* mutant (denoted with the superscript *wox5*) the model reads (it has $W^{wox5} = 0$):

$$\frac{dB^{wox5}}{dt} = P_W(B^{wox5}, 0) - d_B B^{wox5}$$

$$P_B(B^{wox5}, 0) = \alpha\frac{1+\varepsilon_B(K_B B^{wox5})^2}{1+(K_B B^{wox5})^2},$$

$$W^{wox5} = 0,$$

$$P_W(B^{wox5}, 0) = \gamma$$

The model for *bravo* mutant (superscript *bravo*) has $B^{bravo} = 0$ and reads:

$$B^{bravo} = 0,$$

$$P_B(0, W^{bravo}) = \alpha\frac{1+\varepsilon_W(K_W W^{bravo})^2}{1+(K_W W^{bravo})^2},$$

$$\frac{dW^{bravo}}{dt} = P_W(0, W^{bravo}) - d_W W^{bravo},$$

$$P_W(0, W^{bravo}) = \gamma\frac{W_0^2}{W_0^2+(W^{bravo})^2(B_1+1)^2}$$

Finally, for the double *bravo wox5* mutant (superscript *dm*) the model reads:

$$B^{dm} = 0, W^{dm} = 0$$

$$P_B(0,0) = \alpha,\ \ P_W(0,0) = \gamma = P_W(0, W^{bravo})$$

Activation model

For the WT genotype, the activation model reads (see Material and Methods):

$$\frac{dB}{dt} = P_B(B,W) - d_B B$$

$$P_B(B,W) = \alpha \frac{1+\varepsilon_B (K_B B)^2}{1+(K_B B)^2} \frac{1+\varepsilon_W (K_W W)^2}{1+(K_W W)^2}$$

$$\frac{dW}{dt} = P_W(B,W) - d_W W$$

$$P_W(B,W) = \gamma \frac{W_0^2}{W_0^2+W^2}\left(1+\ B_1 \frac{B^2}{B_0^2+B^2}\right)$$

By redefining time as $\tau = td_B$ defining the new nondimensinal variables $b = K_B B, w = K_W W$ and parameters $\tilde{\alpha} = \alpha K_B/d_B$, $\tilde{\gamma} = \gamma K_W/d_W$, $b_0 = B_0 K_B$, $w_0 = W_0 K_W$, we can write a nondimensional version of the model as:

$$\frac{db}{d\tau} = \tilde{\alpha} \frac{1+\varepsilon_B b^2}{1+b} \frac{1+\varepsilon_W w^2}{1+w^2} - b$$

$$\frac{dw}{d\tau} = \frac{d_W}{d_B}\left(\tilde{\gamma} \frac{w_0^2}{w_0^2+w^2}\left(1+\ B_1 \frac{b^2}{b_0^2+b^2}\right) - w\right)$$

With this nondimensional model we compute the distribution of promoter fold-changes when all non-dimensional parameters are allowed to change at once.

For clarity, here we show how to compute the mutants with the full, dimensional model. For the *wox5* mutant the model reads:

$$\frac{dB^{wox5}}{dt} = P_W(B^{wox5}, 0) - d_B B^{wox5}$$

$$P_B(B^{wox5}, 0) = \alpha \frac{1+\varepsilon_B \left(K_B B^{wox5}\right)^2}{1+(K_B B^{wox5})^2},$$

$$W^{wox5} = 0,$$

$$P_W(B^{wox5},0) = \gamma\left(1 + B_1 \frac{(B^{wox5})^2}{B_0^2 + (B^{wox5})^2}\right)$$

The model for *bravo* mutant reads:

$$B^{bravo} = 0,$$

$$P_B(0, W^{bravo}) = \alpha \frac{1+\varepsilon_W(K_W W^{bravo})^2}{1+(K_W W^{bravo})^2},$$

$$\frac{dW^{bravo}}{dt} = P_W(0, W^{bravo}) - d_W W^{bravo},$$

$$P_W(0, W^{bravo}) = \gamma \frac{W_0^2}{W_0^2+(W^{bravo})^2}$$

Finally, for the double *bravo wox5* mutant the model reads:

$$B^{dm} = 0, W^{dm} = 0$$

$$P_B(0,0) = \alpha, \ \ P_W(0,0) = \gamma = P_W(0, W^{Bm})$$

Complex formation model

For the WT genotype, the complex formation model reads (see Material and Methods):

$$\frac{dB}{dt} = \alpha \frac{1+\varepsilon_B(K_B B)^2}{1+(K_B B)^2} \frac{1+\varepsilon_W(K_W W)^2}{1+(K_W W)^2} - \lambda_{BW} BW + \mu_{BW} C_{BW} - \lambda_{BS} BS + \mu_{BS} C_{BS} - d_B B$$

$$\frac{dW}{dt} = \gamma \frac{W_0^2}{W_0^2 + W^2} - \lambda_{BW} BW + \mu_{BW} C_{BW} - \lambda_{WS} WS + \mu_{WS} C_{WS} - d_W W$$

$$\frac{dS}{dt} = \beta - \lambda_{BS} BS + \mu_{BS} C_{BS} - \lambda_{WS} WS + \mu_{WS} C_{WS} - d_S S$$

$$\frac{dC_{BW}}{dt} = \lambda_{BW} BW - \mu_{BW} C_{BW} - d_{BW} C_{BW}$$

$$\frac{dC_{BS}}{dt} = \lambda_{BS} BS - \mu_{BS} C_{BS} - d_{BS} C_{BS}$$

$$\frac{dC_{WS}}{dt} = \lambda_{WS} WS - \mu_{WS} C_{WS} - d_{WS} C_{WS}$$

In the *wox5* mutant the complex formation model reads:

$$\frac{dB^{wox5}}{dt} = \alpha\frac{1+\varepsilon_B(K_B B^{wox5})^2}{1+(K_B B^{wox5})^2} - \lambda_{BS}B^{wox5}S^{wox5} + \mu_{BS}C_{BS}^{wox5} - d_B B^{wox5}$$

$$\frac{dS^{wox5}}{dt} = \beta - \lambda_{BS}B^{wox5}S^{wox5} + \mu_{BS}C_{BS}^{wox5} - d_S S^{wox5}$$

$$\frac{dC_{BS}^{wox5}}{dt} = \lambda_{BS}B^{wox5}S^{wox5} - \mu_{BS}C_{BS}^{wox5} - d_{BS}C_{BS}^{wox5}$$

Therefore, the BRAVO and WOX5 promoters in this mutant are:

$$P_B(B^{wox5},0) = \alpha\frac{1+\varepsilon_B(K_B B^{wox5})^2}{1+(K_B B^{wox5})^2}, \qquad P_W(B^{wox5},0) = \gamma$$

The complex formation model for *bravo* mutant reads:

$$\frac{dW^{bravo}}{dt} = \gamma\frac{W_0^2}{W_0^2+(W^{bravo})^2} - \lambda_{WS}W^{bravo}S^{bravo} + \mu_{WS}C_{WS}^{bravo} - d_W W^{bravo}$$

$$\frac{dS^{bravo}}{dt} = \beta - \lambda_{WS}W^{bravo}S^{bravo} + \mu_{WS}C_{WS}^{bravo} - d_S S^{bravo}$$

$$\frac{dC_{WS}^{bravo}}{dt} = \lambda_{WS}W^{bravo}S^{bravo} - \mu_{WS}C_{WS}^{bravo} - d_{WS}C_{WS}^{bravo}$$

And the promoters in the *bravo* mutant are:

$$P_B(0,W^{bravo}) = \alpha\frac{1+\varepsilon_W(K_W W^{bravo})^2}{1+(K_W W^{bravo})^2}, \qquad P_W(B^{bravo},0) = \gamma\frac{W_0^2}{W_0^2+(W^{bravo})^2}$$

Finally, for the double *bravo wox5* mutant the complex formation model reads (notice that only the factor *S* remains):

$$\frac{dS^{dm}}{dt} = \beta - d_S S^{dm}$$

And the promoters are:

$$P_B(0,0) = \alpha, \;\; P_W(0,0) = \gamma = P_W(0,W^{Bm})$$

Stationary solutions

For each genotype, the stationary solutions are found by imposing the stationarity condition: $\frac{dB}{dt} = 0$ and $\frac{dW}{dt} = 0$, of the equations that describe each genotype.We illustrate this with the alleviation model, the results corresponding to the activation model being computed with the same procedure.

For the WT, when we impose the stationary conditions the following set of two coupled algebraic equations is obtained in the stationary state:

$$d_B B^{WT} = \alpha \left(\frac{1 + \varepsilon_B (K_B B^{WT})^2}{1 + (K_B B^{WT})^2} \right) \left(\frac{1 + \varepsilon_W (K_W W^{WT})^2}{1 + (K_W W^{WT})^2} \right)$$

$$d_W W^{WT} = \gamma \left(\frac{W_0^2}{W_0^2 + (W^{WT})^2 \left(\frac{B_0^2}{B_0^2 + (B^{WT})^2} B_1 + 1 \right)^2} \right)$$

which is solved numerically (see Material and Methods). We denote by B^{WT}, W^{WT} the stationary solutions for the expression of *BRAVO* and *WOX5* in the WT. The stationary *BRAVO* and *WOX5* production activities in the WT are:

$$P_B^{WT} \equiv P_B(B^{WT}, W^{WT}) = \alpha \left(\frac{1 + \varepsilon_B (K_B B^{WT})^2}{1 + (K_B B^{WT})^2} \right) \left(\frac{1 + \varepsilon_W (K_W W^{WT})^2}{1 + (K_W W^{WT})^2} \right)$$

$$P_W^{WT} \equiv P_W(B^{WT}, W^{WT}) = \gamma \left(\frac{W_0^2}{W_0^2 + (W^{WT})^2 \left(\frac{B_0^2}{B_0^2 + (B^{WT})^2} B_1 + 1 \right)^2} \right)$$

where, once we have the stationary values B^{WT}, W^{WT} we can obtain their values by substitution on the above expressions.

We proceed in the same way with each mutant with their corresponding equations set to the stationary state.

For the *wox5* mutant, we have $W^{wox5} = 0$, and the stationary expression of *BRAVO* satisfies

$$B^{wox5} = \frac{\alpha}{d_B} \frac{1+\varepsilon_B (K_B B^{wox5})^2}{1+(K_B B^{wox5})^2}$$

which is solved numerically. The stationary *BRAVO* and *WOX5* promoter activities (productions) in this mutant are:

$$P_B^{wox5} = \alpha \frac{1+\varepsilon_B (K_B B^{wox5})^2}{1+(K_B B^{wox5})^2},$$

$$P_W^{wox5} = \gamma.$$

For the *bravo* mutant in the stationary state we have $B^{bravo} = 0$, and

$$W^{bravo} = \frac{\gamma}{d_W} \frac{W_0^2}{W_0^2 + (W^{bravo})^2 (B_1 + 1)^2}$$

which is solved numerically. Once solved, the stationary promoter activities in this mutant are found as:

$$P_B^{bravo} = \alpha \frac{1+\varepsilon_W (K_W W^{bravo})^2}{1+(K_W W^{bravo})^2},$$

$$P_W^{bravo} = \gamma \frac{W_0^2}{W_0^2 + (W^{bravo})^2 (B_1 + 1)^2}$$

Finally, the model of the double *bravo wox5* mutant already indicates the stationary state values:

$$B^{dm} = 0, W^{dm} = 0$$

$$P_B^{dm} = \alpha, \;\; P_W^{dm} = \gamma = P_W^{wox5}.$$

As indicated above, with the Activation model we proceeded analogously.

The stationary solutions of the complex model were computed by setting the stationarity condition to all variables ($\frac{dB}{dt} = 0, \frac{dW}{dt} = 0, \frac{dS}{dt} = 0, \frac{dC_{BW}}{dt} = 0, \frac{dC_{BS}}{dt} = 0$ and $\frac{dC_{WS}}{dt} = 0$). This results into the following three coupled algebraic equations which need to be solved to find the stationary values of the variables:

$$d_B B^{WT} + \lambda_{BW}\left(1 - \frac{\mu_{BW}}{\mu_{BW} + d_{BW}}\right) B^{WT} W^{WT} + \lambda_{BS}\left(1 - \frac{\mu_{BS}}{\mu_{BS} + d_{BS}}\right) B^{WT} S^{WT}$$

$$= \alpha \frac{1 + \varepsilon_B (K_B B^{WT})^2}{1 + (K_B B^{WT})^2} \frac{1 + \varepsilon_W (K_W W^{WT})^2}{1 + (K_W W^{WT})^2}$$

$$d_W W^{WT} + \lambda_{BW}\left(1 - \frac{\mu_{BW}}{\mu_{BW} + d_{BW}}\right) B^{WT} W^{WT} + \lambda_{WS}\left(1 - \frac{\mu_{WS}}{\mu_{WS} + d_{WS}}\right) W^{WT} S^{WT}$$

$$= \gamma \frac{W_0^2}{W_0^2 + (W^{WT})^2}$$

$$S^{WT} = \frac{\beta}{\lambda_{BS}\left(1 - \frac{\mu_{BS}}{\mu_{BS} + d_{BS}}\right) B^{WT} + \lambda_{WS}\left(1 - \frac{\mu_{WS}}{\mu_{WS} + d_{WS}}\right) W^{WT} + d_S}$$

where the superscript denotes the stationary solution of the wild-type (WT) case. The right-hand side of the two first equations are proportional to the stationary promoter activity of BRAVO and of WOX5 respectively. The stationary heterodimers are given by:

$$C_{BW}^{WT} = \frac{\lambda_{BW}}{\mu_{BW} + d_{BW}} B^{WT} W^{WT}$$

$$C_{BS}^{WT} = \frac{\lambda_{BS}}{\mu_{BS} + d_{BS}} B^{WT} S^{WT}$$

$$C_{WS}^{WT} = \frac{\lambda_{WS}}{\mu_{WS} + d_{WS}} W^{WT} S^{WT}$$

And the stationary productions are:

$$P_B^{WT} \equiv P_B(B^{WT}, W^{WT}) = \alpha \frac{1 + \varepsilon_B (K_B B^{WT})^2}{1 + (K_B B^{WT})^2} \frac{1 + \varepsilon_W (K_W W^{WT})^2}{1 + (K_W W^{WT})^2}$$

$$P_W^{WT} \equiv P_W(B^{WT}, W^{WT}) = \gamma \frac{W_0^2}{W_0^2 + (W^{WT})^2}$$

In the *wox5* mutant the stationary solutions are found by solving:

$$d_B B^{wox5} + \lambda_{BS}\left(1 - \frac{\mu_{BS}}{\mu_{BS} + d_{BS}}\right) B^{wox5} S^{wox5} = \alpha \frac{1 + \varepsilon_B (K_B B^{wox5})^2}{1 + (K_B B^{wox5})^2}$$

$$S^{wox5} = \frac{\beta}{\lambda_{BS}\left(1 - \frac{\mu_{BS}}{\mu_{BS} + d_{BS}}\right)B^{wox5} + d_S}$$

Notice that in this mutant, $W^{wox5} = 0, C_{WB}^{wox5} = 0, C_{WS}^{wox5} = 0$ and the only heterodimer is:

$$C_{BS}^{wox5} = \frac{\lambda_{BS}}{\mu_{BS} + d_{BS}} B^{wox5} S^{wox5}$$

Therefore, the stationary BRAVO and WOX5 productions in the *wox5* mutant are:

$$P_B^{wox5} = \alpha \frac{1 + \varepsilon_B (K_B B^{wox5})^2}{1 + (K_B B^{wox5})^2}$$

$$P_W^{wox5} = \gamma$$

In the *bravo* mutant the stationary solutions are found by solving:

$$d_W W^{bravo} + \lambda_{WS}\left(1 - \frac{\mu_{WS}}{\mu_{WS} + d_{WS}}\right) W^{bravo} S^{bravo} = \gamma \frac{W_0^2}{W_0^2 + (W^{bravo})^2}$$

$$S^{bravo} = \frac{\beta}{\lambda_{WS}\left(1 - \frac{\mu_{WS}}{\mu_{WS} + d_{WS}}\right)B^{bravo} + d_S}$$

Notice that in this mutant, $B^{bravo} = 0, C_{WB}^{bravo} = 0, C_{BS}^{bravo} = 0$ and the only heterodimer is:

$$C_{WS}^{bravo} = \frac{\lambda_{WS}}{\mu_{WS} + d_{WS}} B^{bravo} S^{bravo}$$

Therefore, the stationary BRAVO and WOX5 productions in the *bravo* mutant are:

$$P_B^{bravo} = \alpha \frac{1 + \varepsilon_W \left(K_W W^{bravo}\right)^2}{1 + (K_W W^{bravo})^2}$$

$$P_W^{bravo} = \gamma \frac{W_0^2}{W_0^2 + (W^{bravo})^2}$$

Finally, in the *bravo wox5* double mutant, the stationary solutions are:

$$B^{dm} = 0, W^{dm} = 0, B^{dm} = \frac{\beta}{d_s}, C_{WB}^{dm} = 0, C_{BS}^{dm} = 0, C_{WS}^{dm} = 0$$

and the stationary productions of BRAVO and WOX5 are:

$$P_B^{dm} = \alpha$$

$$P_W^{dm} = \gamma = P_W^{Wm}$$

As indicated, for the complex formation model (as in the alleviation model) the WOX5 production in the *bravo wox5* mutant is the same as in the *wox5* mutant.

In all models, the stationary solutions in the overexpressing lines are computed similarly as in the WT, being the only difference and added constant term in the production as explained in Methods.

APPENDIX FIGURES AND TABLES

Figure S1: **Medial longitudinal view of the *Arabidopsis thaliana* primary root apex.**

Schematic representation of a 6-day-old primary root. At the root apex the stem cell niche is formed by the quiescent center (QC) and the surrounding stem cells, which are highlighted in different colors.

Figure S2: **BRAVO and WOX5 promote primary root growth and lateral root development.**

A) Root length of 6-day-old WT and *bravo-2 wox5-1* mutants in control and after BL treatment (n>30, 3 replicates). Different letters indicate statistically significant differences (p-value < 0.05 Student´s t-test).

B) Lateral root density (number of lateral roots per mm of root length) of 10-day-olf WT, *bravo-2*, *wox5-1* and *bravo-2 wox5-1* mutants (n>52, 3 replicates). Different letters indicate statistically significant differences (p-value < 0.05 Student´s t-test).

Figure S3: BRAVO and WOX5 expression patterns in overexpressing lines.

A) Bars show the relative expression of BRAVO and WOX5 in 35S:WOX5-GR lines when induced with 1µM Dexamethasone for 24 hours. Values in control conditions are not represented as are 1. Data obtained from two independent biological replicates. Asterisks indicate significant differences (* p-value < 0.05, *** p-value < 0.001 Student´s t-test).

B) Bars show the relative expression of BRAVO and WOX5 in 35S:BRAVO-Ei lines when induced with 30 µM β-estradiol for 24 hours. Values in control conditions are not represented as are 1. Data obtained from three independent biological replicates. Asterisks indicate significant differences (** p-value < 0.01 Student´s t-test).

C) Quantification of the GFP fluorescent signal of the roots in D-G. Boxplot indicating the average pixel intensity of the GFP in the stem cell niche. (n>29, 3 biological replicates, Different letters indicate statistically significant differences (p-value < 0.05 Student´s t-test).

D-G) Confocal images of PI-stained 6-day-old roots. GFP-tagged expression is shown in green. pWOX5:GFP in WT and 35S:BRAVO-Ei background in control (D, F) and after 6 days 30 µM β-estradiol induction (E, G). Scale bar: 50 µm.

Figure S4: BRAVO expression in the *bravo wox5* mutant background.

A-D) Confocal images of PI-stained 6-day-old roots. GFP-tagged expression is shown in green. *pBRAVO:GFP* in WT and *bravo-2 wox5-1* background in control (A, C) and after BL treatment (B, D). Scale bar: 50 µm.

E) Quantification of the GFP fluorescent signal of the roots in A-D in the stem cell niche. Different letters indicate statistically significant differences (p-value < 0.05 Student´s t-test). Different confocal settings were used in these images to those in Figures 2 and S5.

Figure S5: BRAVO and WOX5 expression is BL regulated.

A-N) Confocal images of PI-stained 6-day-old roots. GFP-tagged expression is shown in green. **A-C)** *pBRAVO:GFP* in WT, *bravo-2* and *wox5-1* knockout backgrounds in CTL (A-C) and after 48h 4nM BL treatment (D-F). **G-N)** *pWOX5:GFP* in WT, *bravo-2*, *wox5-1* and *bravo-2 wox5-1* knockout backgrounds in CTL (G-J) and after 48h 4 nM BL treatment (K-N). Images in control conditions are the same that are shown in figure 2. Scale bar: 50 µm.

O, P) Quantification of the GFP fluorescent signal of the roots in A-F (O) and G-N (P). Boxplot indicating the average pixel intensity of the GFP in the stem cell niche. (n>25, 3 biological replicates, *p-value < 0.05 Student´s *t*-test for each genotype versus the WT in the same condition). Quantification of lines in control conditions are the same that are shown in figure 2.

These results (together with Figure S4) suggest that the mutual regulation of BRAVO and WOX5, as well as their autoregulation, is not significantly altered by BL treatment.

Figure S6: Biochemical interactions of BRAVO and WOX5 with BES1 and TPL.

A) Yeast two-hybrid assay showing BRAVO interactions with WOX5, BES1 and TPL *in vitro*. In the left column yeast cells were grown on control media, and in the right column yeast cells were grown on control media lacking Leu, Trp and His, indicating an interaction between the proteins. Images in rows 1,3, 8 and 13 are the same that are shown in Figure 4L.

B-D) *In planta* interaction by Bimolecular Fluorescence Complementation assay (BiFC). Confocal images were merged with red fluorescence images corresponding to chlorophyll. Fluorescence was detected 48 h post agroinfiltration. Scale bar: 50 μm. B) BiFC showing BRAVO interaction with BES1 and TPL. Nuclear YFP fluorescence is observed in *N. benthamiana* leaves infiltrated with the BRAVO-eYFPC and both BES1 and TPL-eYFPN constructs. BRAVO-eYFPC and empty-eYFPN are included as a negative control. C) BiFC showing WOX5 interaction with BES1 and TPL. Nuclear YFP fluorescence is observed in *N. benthamiana* leaves infiltrated with the WOX5-eYFPN and both BES1 and TPL-eYFPC constructs. WOX5-eYFPN and empty-eYFPC are included as a negative control. D) BES1-eYFPC and TPL-eYFPN was included as a positive control of interaction. Scale bar: 50 μm.

Figure S7: ROIs used for the quantification of the GFP.

A-B) Confocal images of *pBRAVO:GFP* (A) *and pWOX5:GFP* (B) PI-stained 6-day-old roots. GFP-tagged expression is shown in green. Insets show the GFP channels that were used for the quantification. Only the area inside the yellow circle was used for the GFP quantification.

Figure S8: Promoter activities of BRAVO and WOX5 in all genotypes and overexpressing lines for the alleviation and activation models.

(A,B) Stationary promoter activities for the alleviation (A) and the activation (B) models. To mimic the empirical data, which is noisy, we evaluated the stationary productions pB and pW in each genotype (the WT, each mutant case and each overexpression line), n times each, setting a different set of parameter values each time, and for each genotype. We reasoned that the noise found in the empirical data could be mimicked by small variations in parameter values, and set a variation of 30% around a default value (parameter set values were chosen randomly from a uniform distribution between $P_0/1.3$ and $1.3P_0$, where P_0 are the default values of parameter of the dimensional models (see their values in Table S1 and Materials and Methods)). We used n=1000. This figure is to be compared with Figures 2H and S4E, that are the corresponding experimental counterparts (recall vertical scales are arbitrary). The results for the WT, the mutants and the overexpressing lines correspond to different set of parameter values. Thus, each circle herein mimics a single root. Notice that the procedure and the interpretation is distinct from Figure 3C,D, Appendix Figures S9 and S10.

(C-F) Cartoon (C,D) and simulation results (E,F) of the alleviation (C,E) and activation (D,F) models as a function of the control parameter x. This control parameter increases WOX5 and reduces BRAVO promoter activities (blue and red triangles; according to $\alpha=0.3/x$, $\gamma=250x/(x+9)$). $x=1$ corresponds to the CTL condition, while $x>1$ can mimic growth under Brassinolide conditions (green shaded area). The experimentally observed values in CTL conditions are computed as ratios of the mean GFP (the same values as in Figure 3C,D) and are drawn as red and blue squares. Error bars of these data are not depicted for clarity. In the plots (E,F), the region of fold change FC<1 (i.e. the promoter activity is reduced in the mutant) is shaded in gray to visually distinguish it from the region where FC>1 (i.e. the promoter activity is increased in the mutant). Notice that based on the equations of the alleviation model (Eqs. 1-4 in Material and Methods), regulation of WOX5 by BRAVO is set through WOX5 and therefore the following equality always holds $pW^{wox5} = pW^{dm}$ and hence $\frac{pW^{wox5}}{pW^{\mathrm{WT}}} = \frac{pW^{dm}}{pW^{\mathrm{WT}}}$. In contrast, this equality does not hold in the activation model. For BRAVO promoter, and since BRAVO is set to self-repress,

$pW^{wox5} \neq pW^{dm}$ and hence $\frac{pB^{wox5}}{pB^{\mathrm{WT}}} \neq \frac{pB^{dm}}{pB^{\mathrm{WT}}}$ in both models, albeit they can take similar values.

Figure S9: The effects of varying the model's parameters on the promoter fold-changes for the alleviation and activation models.

Boxplots showing the distribution of promoter pBRAVO and pWOX5 fold-changes in each mutant and overexpression lines over the WT obtained in the alleviation (left panels) and activation models (right panels), for a parameter space exploration larger than in Figure 3C,D and for exploration of each parameter individually (A,B). The results are obtained following the same procedure as in Figure 3C,D but for a random distribution of parameter values between $P_0/10$ and $10P_0$, where P_0 is the default set of non-dimensional parameter values (see Table S1). As in Figure 3C,D, for each run (i.e. each set of random parameter values), the WT, mutants and overexpressor cases are computed all with the same parameter values. Circles denote the fold-change obtained in each run, which indicates the mean fold-change predicted by the model for that parameter set.

A, B) The effect of each parameter is depicted. The results correspond to the fold-changes obtained when only one parameter is randomly changed, within a range of a tenth and ten times their default values (see Table 1 in Supplementary Text). The parameter that is changed is indicated in each panel.

C, D) The effect of changing randomly all the parameters simultaneously. This is analogous to Figure 3C, D, but for a wider range of the parameter change.

Figure S10: The formation of a complex between BRAVO and WOX5 can be interpreted as an alleviation mechanism.

A) The formation of the BRAVO-WOX5 complex, together with the depicted transcriptional regulation (BRAVO and WOX5 self-inhibitions and WOX5 activates BRAVO) can account for the trends of fold-changes found experimentally. The model here only considers BRAVO and WOX5 proteins and the complex they form. The alleviation mechanism is exemplified by the decrease of WOX5 promoter in the *bravo* mutant. This decrease is absent if the BRAVO-WOX5 complex can not form (B).

B) The same transcriptional regulations as in A but without the formation of the BRAVO-WOX5 complex. Without the formation of a complex, the model cannot explain all the fold-changes seen experimentally. Specifically, WOX5 promoter does not change in the *bravo* mutant, denoting absence of regulation of *WOX5* by BRAVO.

C) Introducing additional elements (*S*) capable of binding to BRAVO and WOX5 is still compatible with the experimental fold-changes in promoter expression. Comparison with A shows that the competing factor *S* reduces the effect of overexpression of BRAVO on *WOX5* expression.

D, E) If the additional factors are only able to bind to either BRAVO or WOX5, their mutual cross-regulations may become impaired. In the case of BRAVO (D) the effect is more severe as it is in very low quantities, allowing S to sequester it and impede its function (hence fold-changes in the *bravo* mutant are 1). If S only binds to WOX5 (E) the effect is less dramatic, as WOX5 remains at high enough levels to perform its function.

F,G) Fold-changes of the concentration of BRAVO in the *wox5* mutant with respect to the WT (F) and of WOX5 in the *bravo* mutant with respect to the WT (G) for different values of binding strengths with the additional factor *S*, showing that if both BRAVO and WOX5 are able to bind to *S* (panel F, dark pink zone), the fold-change of BRAVO in the *wox5* mutant can be large (particularly, BRAVO levels decrease). Notice that BRAVO concentration is the concentration of BRAVO protein that is not bound to WOX5 nor to *S*. Similarly, WOX5 concentration is the concentration of WOX5 protein that is not bound to BRAVO nor to *S*. If there were no competing factor, BRAVO concentration could increase in the *wox5* mutant and WOX5 concentration could

increase in the *bravo* mutant despite their expressions would decrease in both cases. This is because in the WT the proteins would be more produced but more sequestered between them. The competing factor S enables that in the single mutants, S sequesters even further than in the WT either BRAVO or WOX5.

Panels (A-E) each depict a cartoon of the regulatory interactions of the scenario studied and the boxplots of the distribution of BRAVO and WOX5 promoter fold-changes in each mutant and overexpression lines over the WT. These boxplots are obtained by solving the corresponding model equations (Eqs. 6-11) in the stationary state for N=1000 different sets of parameter values, following the same procedure as in Figure 3C,D: The parameter sets are randomly chosen from uniform distributions of each parameter over the range $P_0/2$ and $2P_0$, where P_0 is the default set of non-dimensional parameters (see Table S1). For each run, the WT, mutants and overexpressor cases are computed all with the same parameter values. Circles denote the fold-change obtained in each run, which indicates the mean fold-change predicted by the model for that parameter set. All parameters, except for degradations, unbinding coefficients and the parameters that define each scenario, are randomly changed between each set. The parameters that define the scenario and are unchanged are: In A, $\beta = 0$ (no production of S) as well as all parameters related to S are set to zero. In B, as in A, and additionally $\lambda_{BW} = \mu_{BW} = d_{BW} = 0$ so that BRAVO-WOX5 complex does not exist. In D, $\lambda_{WS} = \mu_{WS} = d_{WS} = 0$, such that no WOX5-S complex exists. In E, $\lambda_{BS} = \mu_{BS} = d_{BS} = 0$ and hence no BRAVO-S complex exists.

Table S1. Parameter values for the mathematical models.

Parameter values used to perform the numerical simulations. All are in arbitrary units. The right-most column indicates the concentration and time scales in which these values could be meaningful in a biological context.

Table S2. List of plant material lines used in this study.

Table S3. List of primers used in this study.

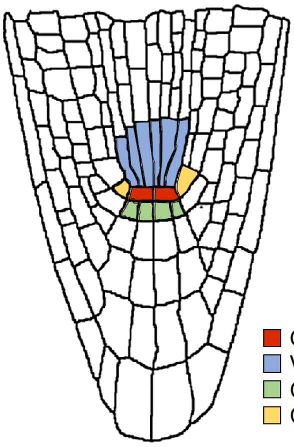

Figure S1: Medial longitudinal view of the *Arabidopsis thaliana* primary root apex.

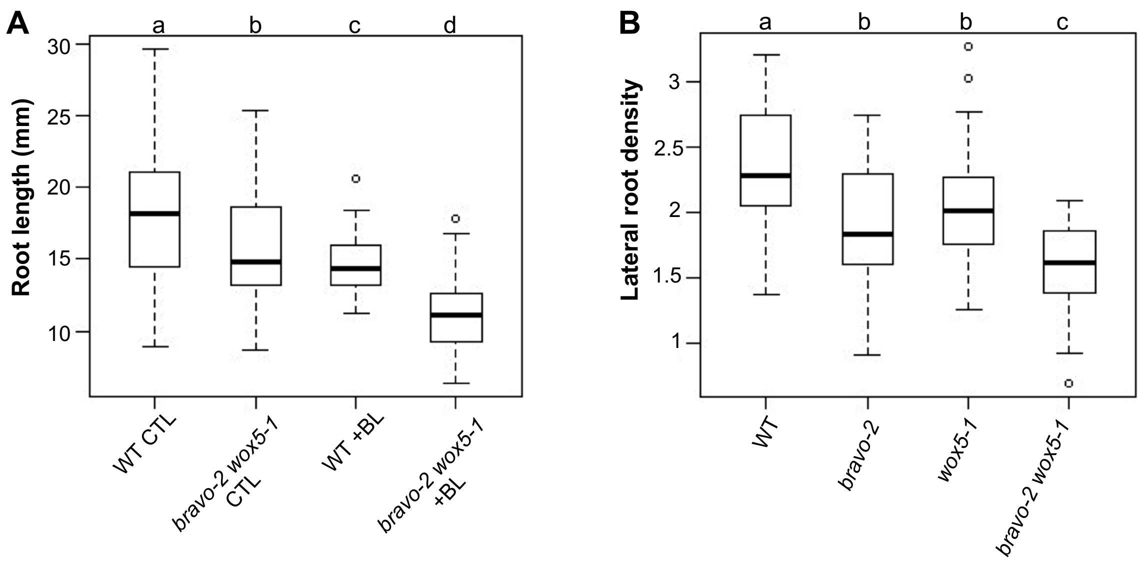

Figure S2: **BRAVO and WOX5 promote primary root growth and lateral root development.**

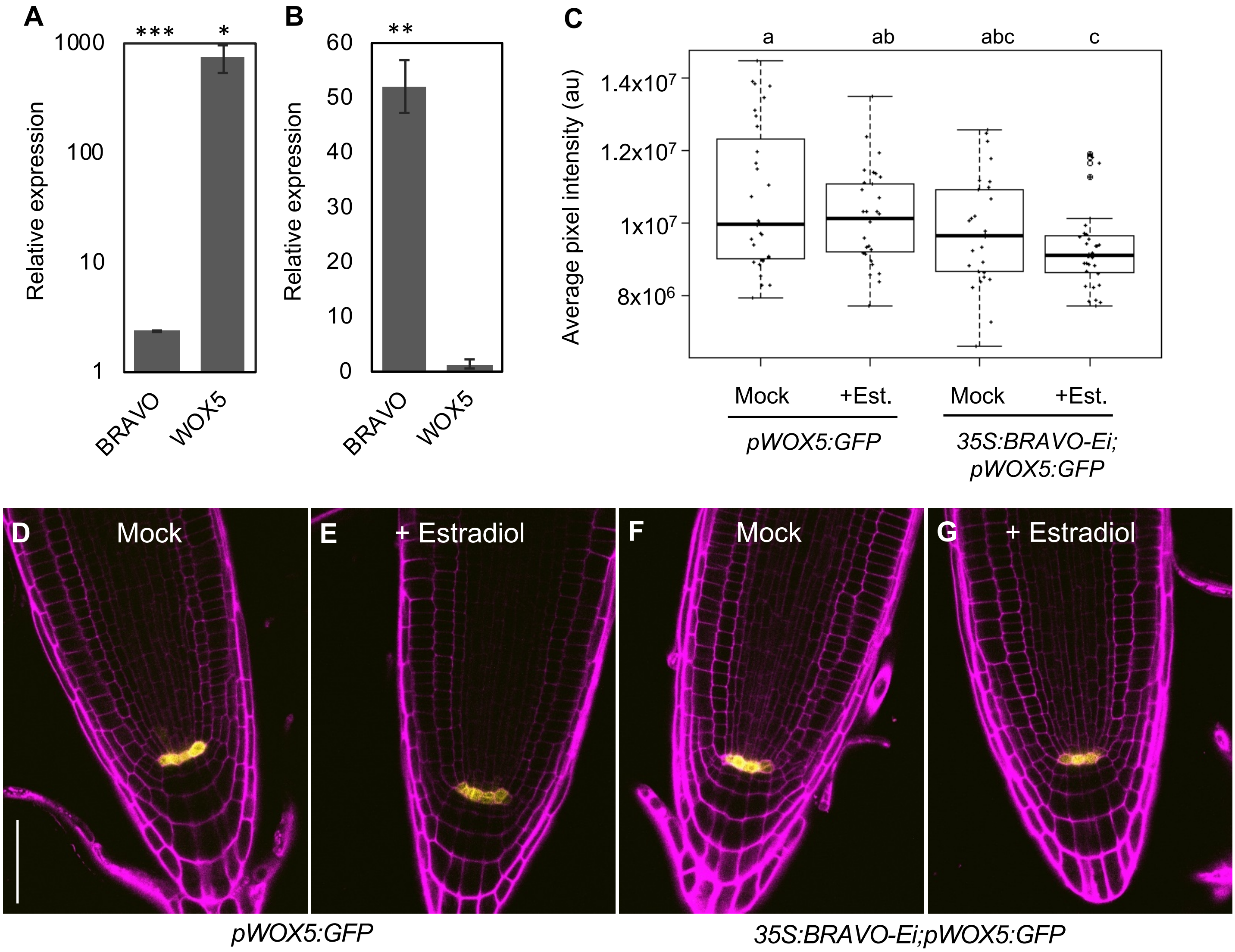

Figure S3: BRAVO and WOX5 expression patterns in overexpressing lines.

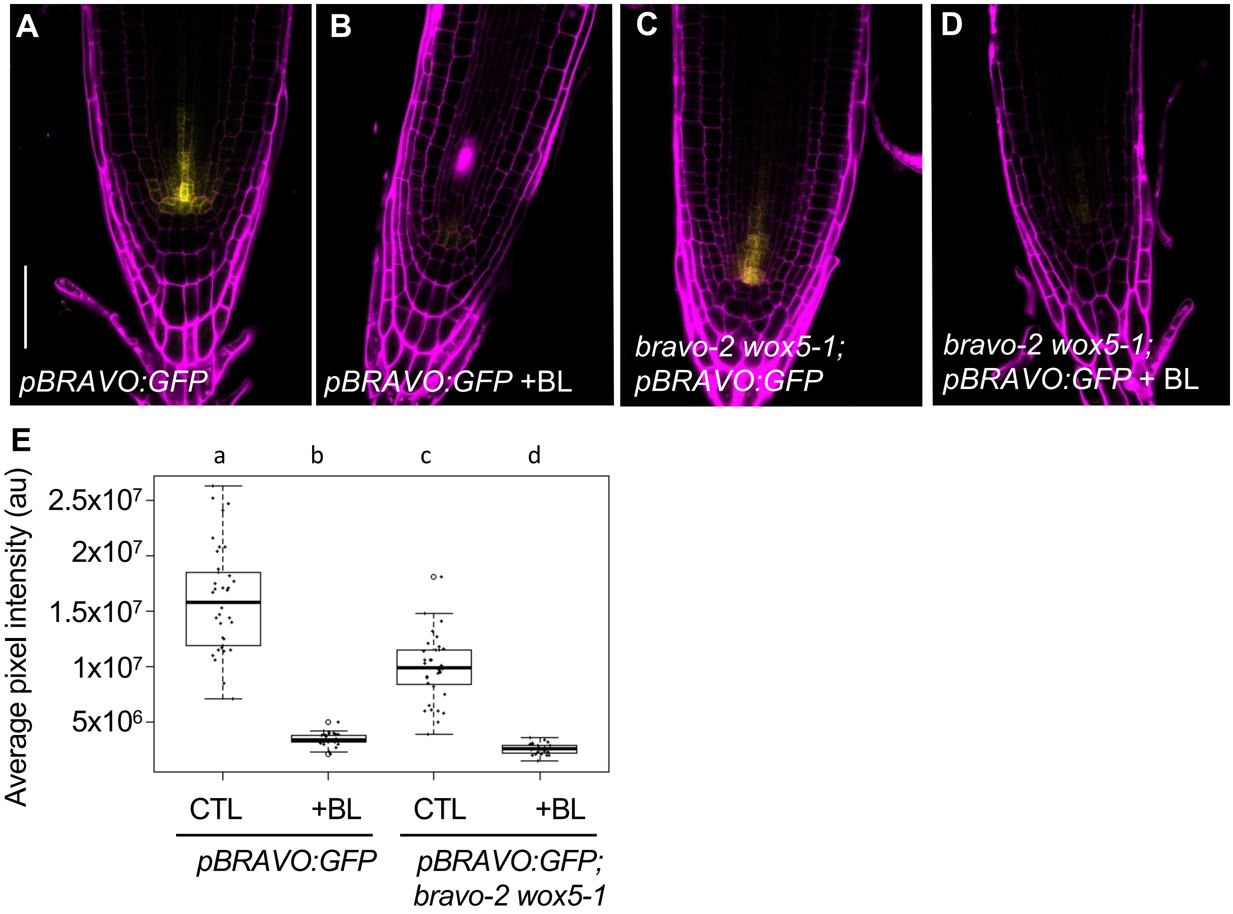

Figure S4: BRAVO expression in the *bravo wox5* mutant background.

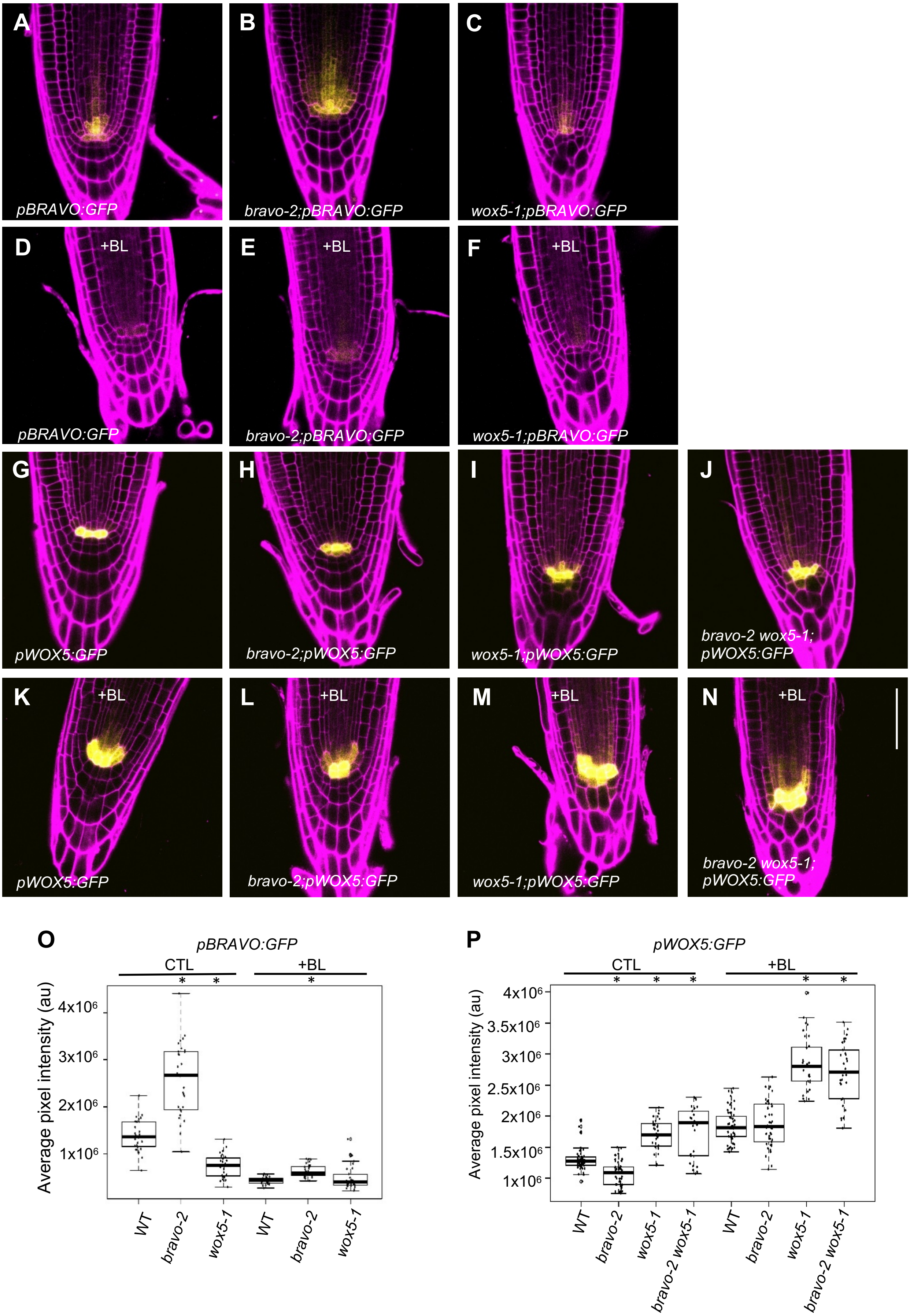

Figure S5: BRAVO and WOX5 expression is BL regulated.

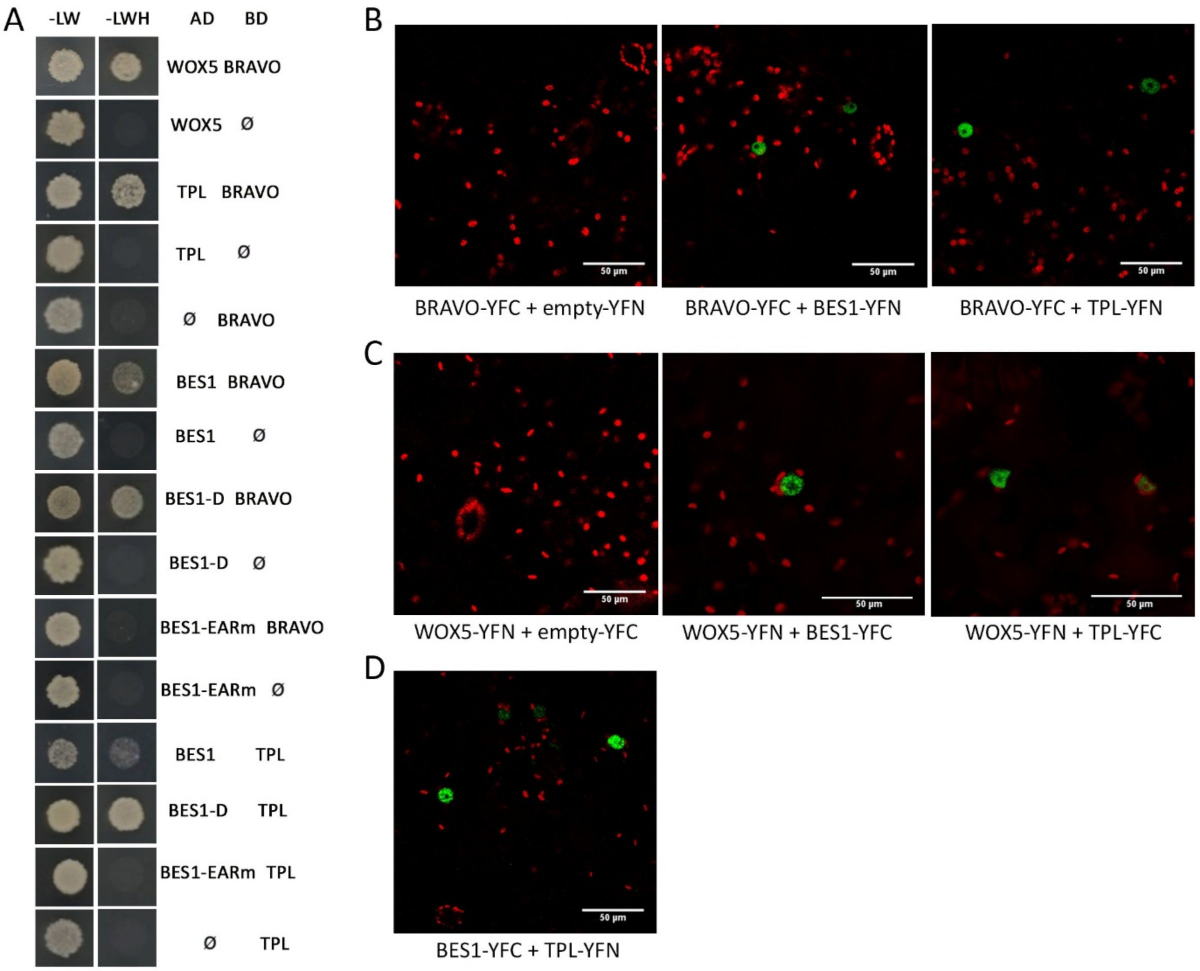

Figure S6: Biochemical interactions of BRAVO and WOX5 with BES1 and TPL.

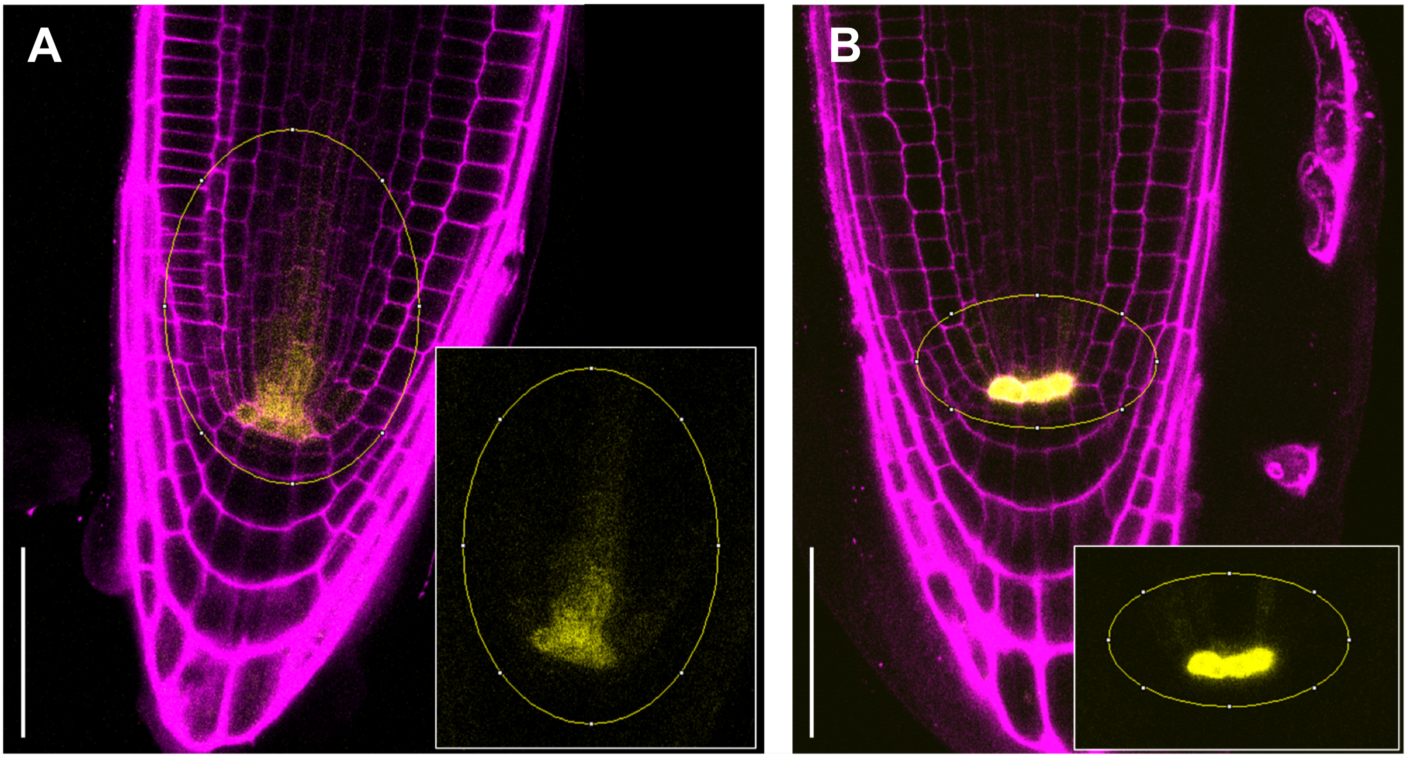

Figure S7: ROIs used for the quantification of the GFP.

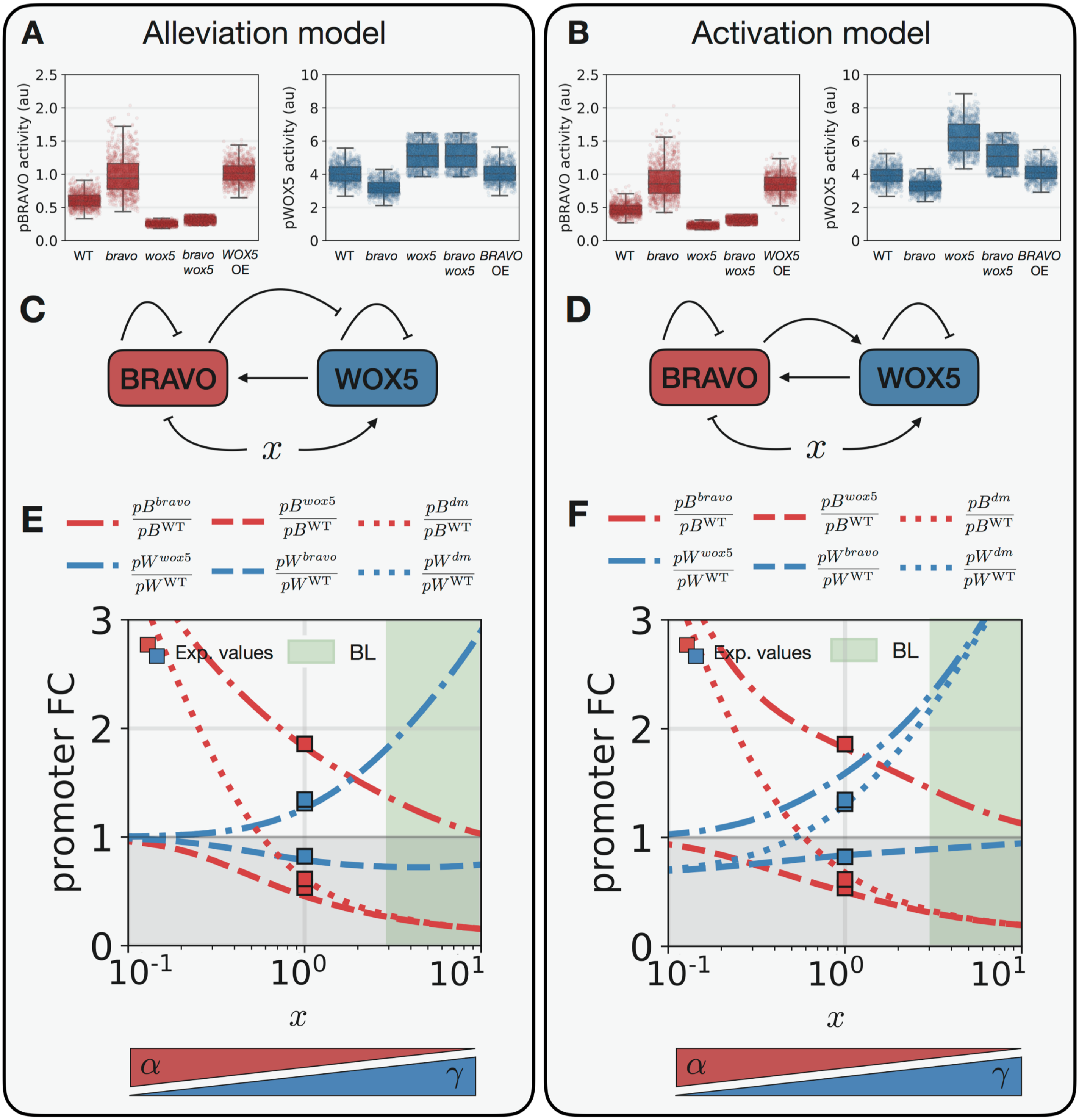

Figure S8: Promoter activities of BRAVO and WOX5 in all genotypes and overexpressing lines for the alleviation and activation models.

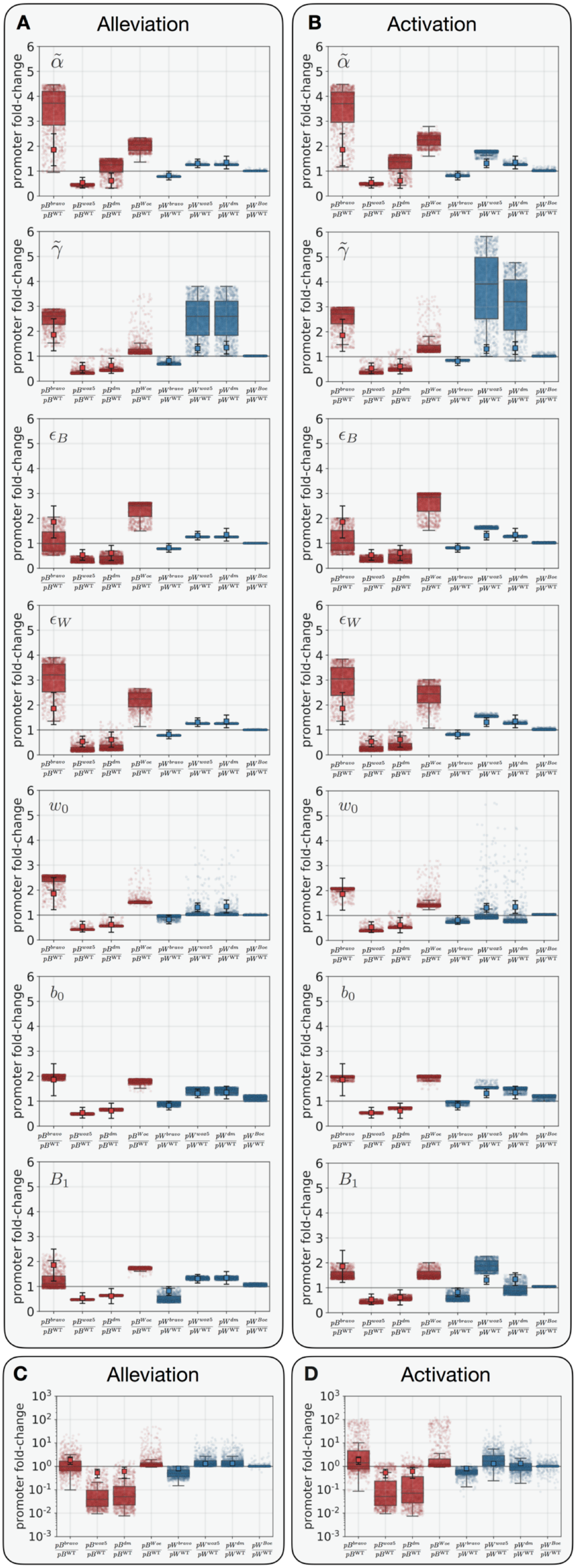

Figure S9: The effects of varying the model's parameters on the promoter fold-changes for the alleviation and activation models.

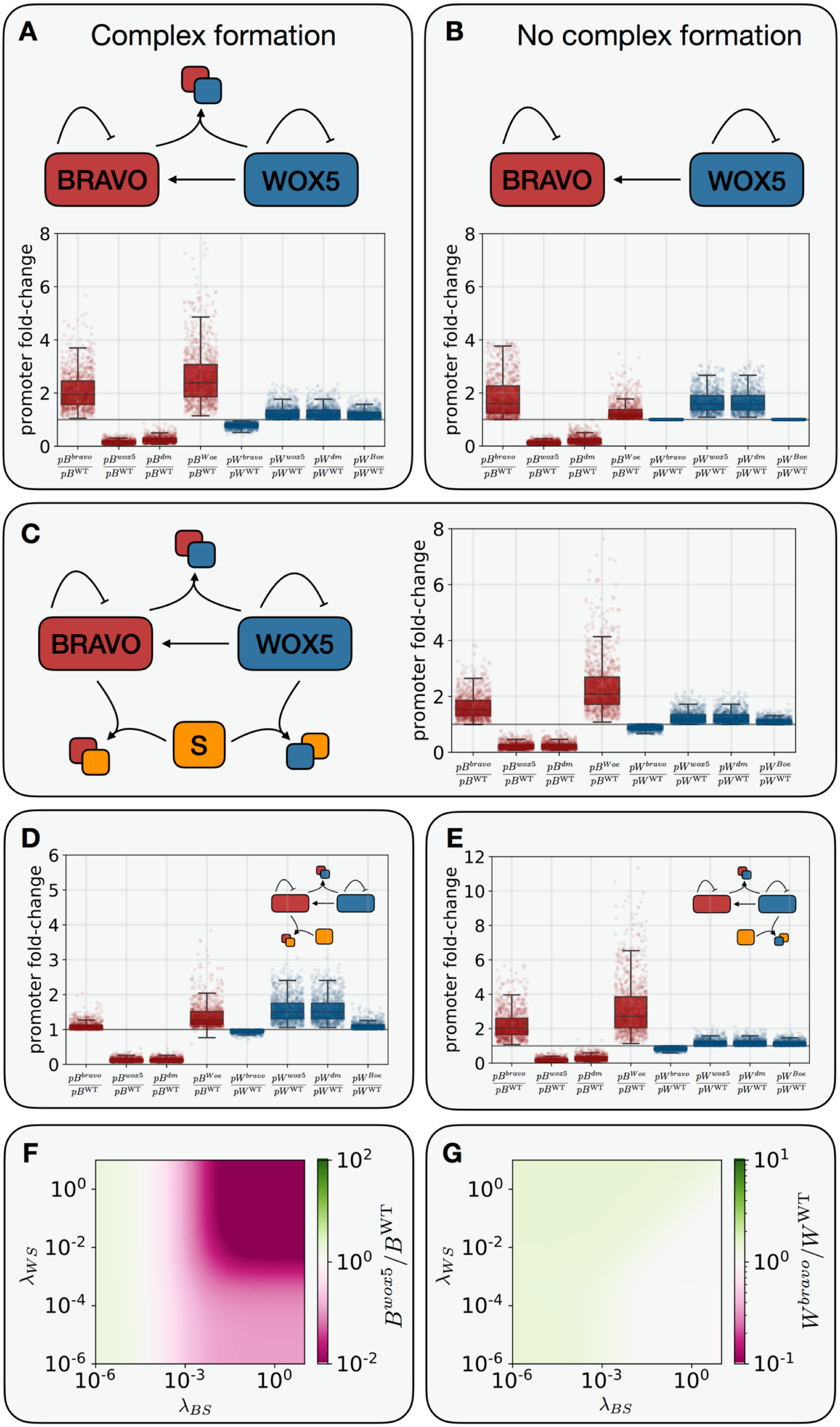

Figure S10: The formation of a complex between BRAVO and WOX5 can be interpreted as an alleviation mechanism.

Table S1. Parameter values for the mathematical models.

Parameter values used to perform the numerical simulations. All are in arbitrary units. The right-most column indicates the concentration and time scales in which these values could be meaningful in a biological context.

**Alleviation model**		
**Parameter**	**Value**	**Units**
α	0.3	nM/min
γ	5	nM/min
K_B	0.02	nM^{-1}
K_W	0.0017	nM^{-1}
ε_B	0.15	-
ε_W	10	-
W_0	800	nM
B_0	10	nM
B_1	1	-
d_B	0.01	min^{-1}
d_W	0.01	min^{-1}
A_0	10	-
G_0	10	-

**Activation model**		
**Parameter**	**Value**	**Units**
α	0.3	nM/min
γ	5	nM/min
K_B	0.03	nM^{-1}
K_W	0.0015	nM^{-1}
ε_B	0.15	-
ε_W	10	-
W_0	430	nM
B_0	25	nM
B_1	0.5	-
d_B	0.01	min^{-1}
d_W	0.01	min^{-1}
A_0	10	-
G_0	10	-

**Complex formation model**		
**Parameter**	**Value**	**Units**
α	0.5	nM/min
γ	5	nM/min
β	1	nM/min
K_B	0.1	nM^{-1}
K_W	0.005	nM^{-1}
ε_B	0.5	-
ε_W	10	-
W_0	415	nM
d_B	0.01	min^{-1}
d_W	0.01	min^{-1}
d_S	0.01	min^{-1}
λ_{BW}	0.01	$nM^{-1}min^{-1}$
λ_{BS}	0.01	$nM^{-1}min^{-1}$
λ_{WS}	0.01	$nM^{-1}min^{-1}$
μ_{BW}	0.02	min^{-1}
μ_{BS}	0.01	min^{-1}
μ_{WS}	0.01	min^{-1}
d_{BW}	0.01	min^{-1}
d_{BS}	0.01	min^{-1}
d_{WS}	0.01	min^{-1}
A_0	10	-
G_0	10	-

**Alleviation model (adimensional)**	
**Parameter**	**Value**
$\tilde{\alpha}$	0.9
$\tilde{\gamma}$	0.85
ε_B	0.15
ε_W	10
w_0	1.36
b_0	0.3
B_1	1
A_0	10
G_0	10

**Activation model (adimensional)**	
**Parameter**	**Value**
$\tilde{\alpha}$	0.9
$\tilde{\gamma}$	0.75
ε_B	0.15
ε_W	10
w_0	0.65
b_0	0.75
B_1	0.5
A_0	10
G_0	10

Table S2. List of plant material lines used in this study.

Name	Description	Reference
bravo-2	Knock out mutant	Vilarrasa-Blasi *et al.,* 2014
wox5-1	Knock out mutant	Sarkar *et al.*, 2007
35S:WOX5-GR	Overexpressor mutant	Sarkar *et al.*, 2007
35S:BRAVO-Ei	Overexpressor mutant	Vilarrasa-Blasi *et al.,* 2014
pBRAVO:GFP	Promoter reporter line	Lee *et al.*, 2006
pWOX5:GFP	Promoter reporter line	Sarkar *et al.*, 2007

Table S3. List of primers used in this study.

Name	Sequence (5'-3')	Description
bravo-2 F	TCCCTTAATCCCTAAACCCAGC	Genotype bravo mutation Forward primer
bravo-2 R	CCTGATGCAAGGGTACTATCG	Genotype bravo mutation Reverse primer
wox5-1 F	ATCTCATAAACCATGCATCGG	Genotype wox5 mutation Forward primer
wox5-1 R	TCGCTGGTTCCGATATACAAC	Genotype wox5 mutation Reverse primer
LBb1.3	ATTTTGCCGATTTCGGAAC	T-DNA border primer
RT-ACTIN2 F	CTGGATCGGTGGTTCCATTC	ACTIN2 RT-qPCR Forward primer
RT-ACTIN2 R	CCTGGACCTGCCTCATCATAC	ACTIN2 RT-qPCR Reverse primer
RT-BRAVO F	TGTTAGCAGCTCATCGAGCCT	BRAVO RT-qPCR Forward primer
RT-BRAVO R	GATGACGTGCCAATGGTTCTT	BRAVO RT-qPCR Reverse primer
RT-WOX5 F	TGATCTGTTTCGAGCCGGTC	WOX5 RT-qPCR Forward primer
RT-WOX5 R	AAACATTCTTGCTCTCTATCTTGCC	WOX5 RT-qPCR Reverse primer
